# Activation of the two microRNA clusters C19MC and miR-371-3 does not play prominent role in thyroid cancer

**DOI:** 10.1186/1755-8166-5-40

**Published:** 2012-10-15

**Authors:** Volkhard Rippe, Inga Flor, Johannes Wolfram Debler, Norbert Drieschner, Birgit Rommel, Daniel Krause, Klaus Junker, Jörn Bullerdiek

**Affiliations:** 1 Center for Human Genetics, University of Bremen, Leobener Str. ZHG, 28359, Bremen, Germany; 2 Department of Pathology, Hospital Bremen-Mitte, St.-Jürgen-Str. 1, 28177, Bremen, Germany

**Keywords:** Thyroid tumors, microRNAs, C19MC, miR-371-3

## Abstract

Chromosomal rearrangements of band 19q13.4 are frequent cytogenetic alterations in benign thyroid adenomas. Apparently, these alterations lead to the upregulation of genes encoding microRNAs of two clusters mapping to the breakpoint region, i.e. miR-371-3 and C19MC. Since members of both clusters have been associated with neoplastic growth in other tumor entities the question arises whether or not their upregulation predisposes to malignant transformation of follicular cells of the thyroid. To address this question we have quantified the expression of miR-372 and miR-520c-3p in samples of 114 thyroid cancers including eight anaplastic thyroid carcinomas, 25 follicular thyroid carcinomas, 78 papillary thyroid carcinomas (including 13 follicular variants thereof), two medullary thyroid carcinomas and one oncocytic thyroid carcinoma. Additionally, we quantified miR-371a-3p and miR-519a-3p in selected samples. While in neither of the cases miR-520c-3p and miR-519a-3p were found to be upregulated, one papillary and one anaplastic thyroid carcinoma, respectively, showed upregulation of miR-372 and miR-371a-3p. However, in these cases fluorescence in situ hybridization did not reveal rearrangements of the common breakpoint region as affected in adenomas. Thus, these rearrangements do apparently not play a major role as first steps in malignant transformation of the thyroid epithelium. Moreover, there is no evidence that 19q13.4 rearrangements characterize a subgroup of thyroid adenomas associated with a higher risk to undergo malignant transformation. Vice versa, the mechanisms by which 19q13.4 rearrangements contribute to benign tumorigenesis in the thyroid remain to be elucidated.

## Background

MicroRNAs (miRNAs) are short non protein-coding RNA molecules that negatively regulate gene expression by binding to the 3' UTR of mRNA thereby inhibiting its transcription or promoting nucleolytic cleavage. Abnormal expression patterns of miRNAs have been found in many types of tumors and, as to malignant tumors, are able to promote cancer specific properties like metastasis, angiogenesis or proliferation [1,2].

We have been able to show that the most frequent recurrent chromosomal rearrangement in benign thyroid tumors, i.e. translocations of chromosomal band 19q13, initially described more than 20 years ago [3], clusters within a 150 kb region assigned to 19q13.4 [4]. More recently, we have demonstrated that these aberrations target the two miRNA gene clusters C19MC (chromosome 19 microRNA cluster) and miR-371-3 resulting in their transcriptional activation likely due to juxtaposing them to transcriptional activators [5] or by removal of CpG rich regions [6] upstream of the clusters.

Normally, the expression of miRNAs of both clusters is abundant in embryonic stem cells (ESC) [7-12] and later becomes repressed in the embryo whereas expression of both clusters persists in the placenta until birth [13,14]. Transcriptional activity of C19MC results from the paternal allele only because the maternal allele is silenced by methylation [15]. However, the biological function of these miRNAs for the placenta development is not yet understood. In a recent set of interesting papers on miRNAs from the miR-371-3 cluster in tumors, their overexpression has been correlated with an invasive cellular behavior [16-19]. On the other hand, miRNAs of C19MC have been described to have oncogenic as well as tumorsuppressive characteristics [for summary see [20]].

Nevertheless, it has not yet been tested if, akin to adenomas with 19q13.4 rearrangements, subsets of thyroid carcinomas also overexpress the genes of both clusters which may indicate that those malignant tumors are derived from pre-existing benign lesions. We therefore examined 114 formalin-fixed, paraffin-embedded (FFPE) tumor samples of the major histological groups of thyroid cancer for their expression of miR-372 and miR-520c-3p, respectively. Selected samples were also analyzed for their expression of miR-371a-3p and miR-519a-3p. We found no cases expressing both miRNAs within the range observed in adenomas with 19q13.4 rearrangements. Interestingly though, two tumors expressed miR-372 and miR-371a-3p at a comparable level.

## Results

By quantitative real-time PCR we first measured miR-372 levels in 114 thyroid carcinomas of different histological groups. Our samples included eight anaplastic thyroid carcinomas (ATC), 25 follicular thyroid carcinomas (FTC), 78 papillary thyroid carcinomas (PTC), including 13 follicular variants of PTC, two medullary thyroid carcinomas (MTC) and one oncocytic thyroid carcinoma (OTC). As a subgroup of thyroid adenomas is shown to overexpress both clusters due to chromosomal rearrangements involving chromosomal band 19q13.4 [5] we used a thyroid adenoma sample (S1016) of this subgroup as a positive control for quantification of miR-372, respectively.

**Figure 1:**
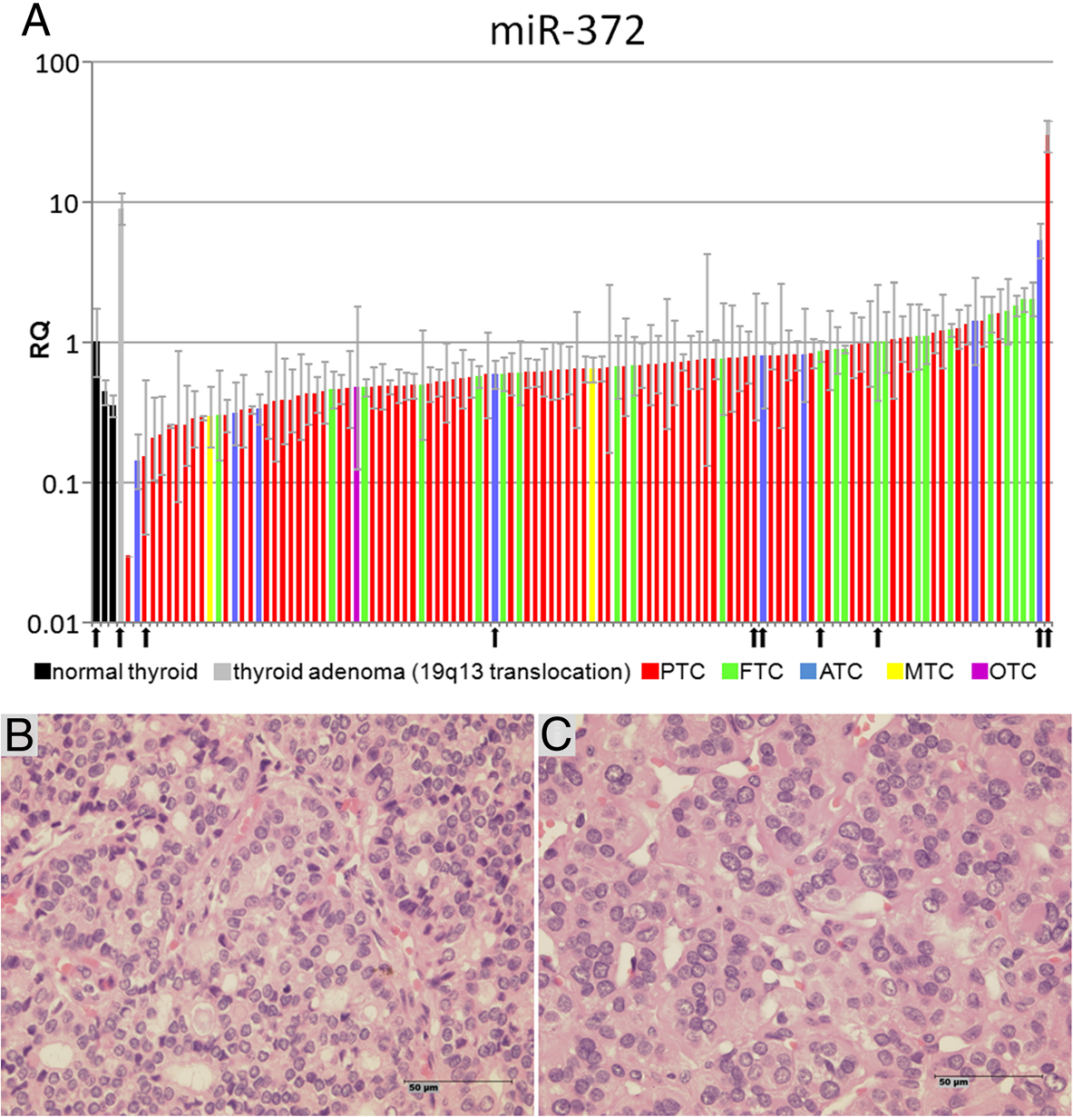
**miR-372 was upregulated in 2 of 114 thyroid carcinomas.** (**A**) Relative expression of miR-372 was quantified in 114 thyroid carcinoma samples of different types (PTC: papillary thyroid carcinoma, FTC: follicular thyroid carcinoma, ATC: anaplastic thyroid carcinoma, MTC: medullary thyroid carcinoma, OTC: oncocytic thyroid carcinoma). Expression is compared to three different normal thyroid tissues. The thyroid tissue showing the highest expression of miR-372 was used as calibrator. A thyroid adenoma with 19q13.4 rearrangement (S1016) served as a positive control of abundant miR-372 expression. The arrows indicate the samples that also appear in Figure 3 (from left to right: normal thyroid tissue, S1016, KS 99, KS 97, KS 61, KS 41, KS 82, KS 106, KS 100, KS 13). RQ: relative quantity (logarithmic scale). The lower panel shows histological sections of (**B**) case KS 13, a follicular variant of papillary thyroid carcinoma (PTC) and (**C**) case KS 100, an anaplastic thyroid carcinoma (ATC).

Compared to normal thyroid tissue miR-372 was clearly upregulated in only two tumors (KS 13 and KS 100) (2.4%) (Figure 1A). KS 13 is a follicular variant of a PTC and KS 100 is an ATC (Figure 1B, C).

**Figure 2:**
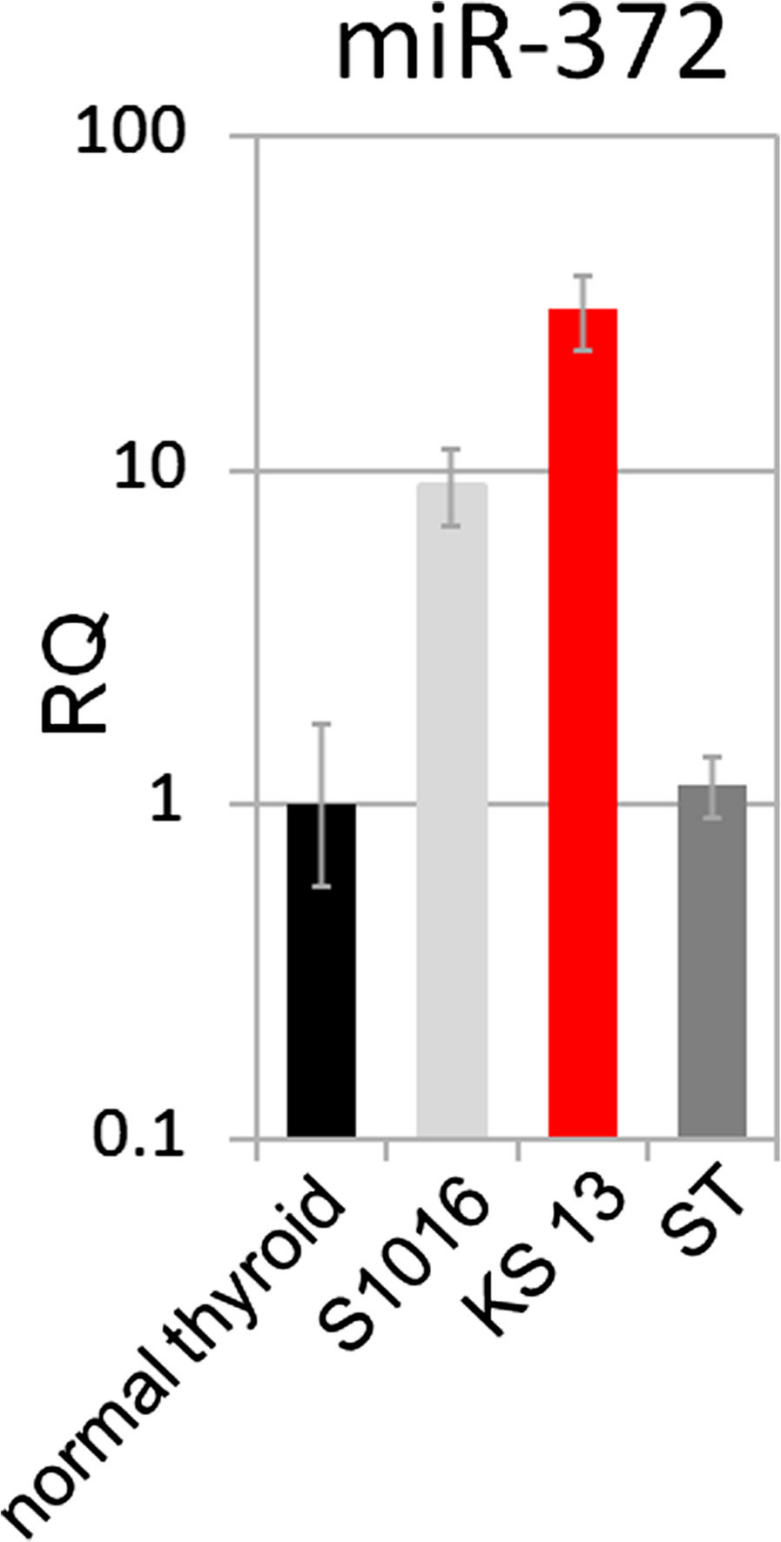
**Relative expression of miR-372 in case KS 13 and surrounding normal tissue.** Surrounding normal tissue (ST) was obtained from another FFPE block of the same sample. Expression is compared to normal thyroid tissue and a thyroid adenoma with 19q13.4 rearrangement (S1016) as positive control.

In order to validate that overexpression of miR-372 is indeed tumor-specific we used another FFPE block of case KS 13 to separate the surrounding tissue from the tumor. Overexpression of miR-372 was clearly confined to the tumor and absent in the surrounding normal thyroid tissue (Figure 2).

**Figure 3:**
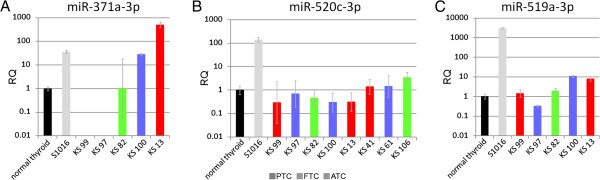
**Relative expression of miR-371a-3p, miR-520c-3p, and miR-519a-3p in thyroid carcinoma samples.** (**A**) Relative expression of miR-371a-3p was quantified in five thyroid carcinoma samples of different histological subgroups. (**B**) Relative expression of miR-520c-3p was quantified in 114 thyroid carcinoma samples of different types (as no upregulation was observed in any of the samples only a selection of samples is shown including the three cases with the highest expression of miR-520c-3p. (**C**) Relative expression of miR-519a-3p in five thyroid carcinoma samples of different types. In A,B and C the expression is compared to normal thyroid tissue and a thyroid adenoma with 19q13.4 rearrangement (S1016) as positive control. RQ: relative quantity (logarithmic scale). PTC: papillary thyroid carcinoma, FTC: follicular thyroid carcinoma, ATC: anaplastic thyroid carcinoma.

To further corroborate our results we next quantified another member of miR-371-3 (i.e. miR-371a-3p) in KS 13 and KS 100 and in one sample of each major histological subgroup (ATC, PTC, FTC), respectively. Akin to what was observed for miR-372, miR-371a-3p was upregulated in KS 13 and KS 100 with expression levels similar to or clearly exceeding that of the positive control S1016 whereas miR-371a-3p was not upregulated in the three other samples (Figure 3A).

We then focused on the cluster C19MC and quantified the expression of miR-520c-3p in all of the 114 thyroid carcinoma samples and miR-519a-3p in the same samples that were used to quantify miR-371a-3p. Neither miR-520c-3p nor miR-519a-3p were upregulated in any of the samples but expressed at a level comparable to that of the normal thyroid tissue; at most, a slight increase could be noted for miR-519a-3p in KS 13 and KS 100 which, however, did not reach levels comparable to that of the thyroid adenoma with 19q13.4 rearrangement (Figure 3B,C).

**Figure 4:**
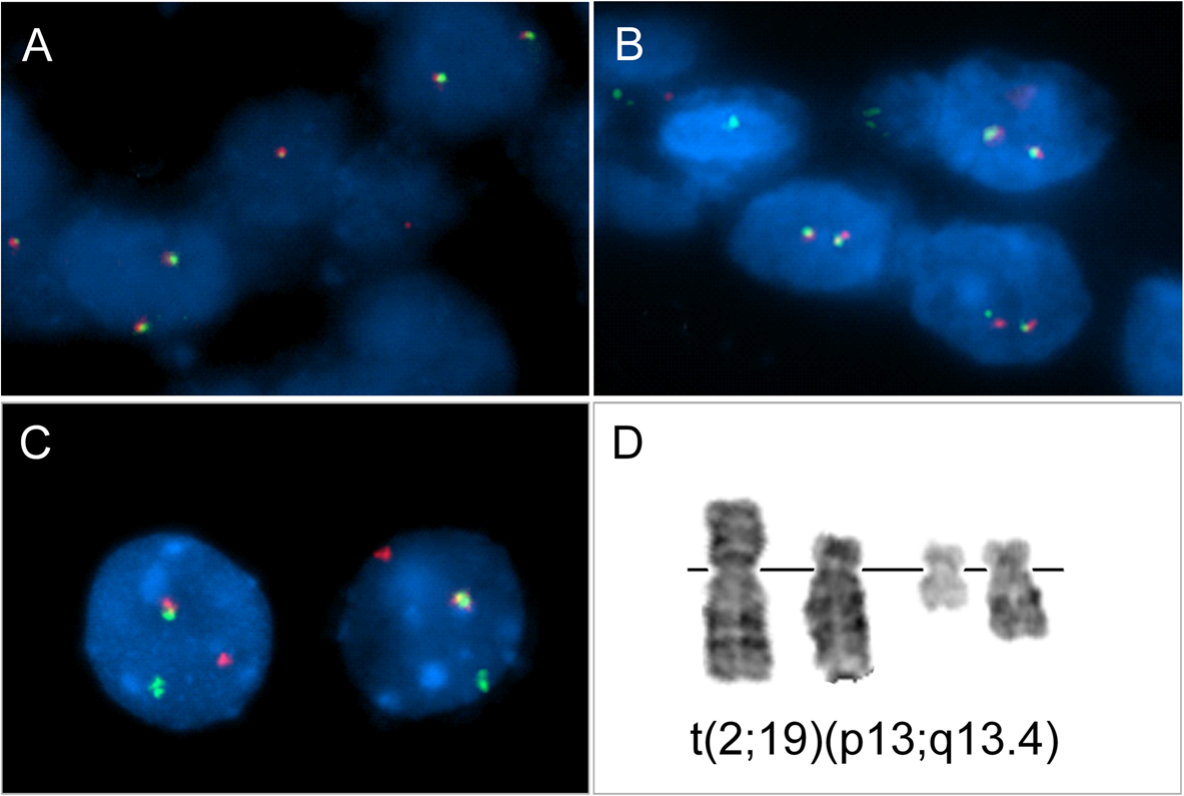
**Fluorescence in situ hybridization for the detection of 19q13.4 rearrangements**. Interphase fluorescence in situ hybridization (FISH) was carried out with a dual-color break-apart rearrangement probe (TBPC19; thyroid adenoma breakpoint cluster 19q13.4) for nuclei of cases KS 13 (**A**), KS 100 (**B**), and the positive control S1016 (**C**), respectively. In addition, a partial karyotype of the latter case with an apparently balanced translocation t(2;19)(p13;q13.4) is shown (**D**).

In order to check whether the two samples KS 100 and KS 13 showing an increased expression of miR-372 and miR-371a-3p harbor 19q13.4 rearrangements we performed fluorescence in situ hybridization (FISH) with a dual-color break-apart rearrangement probe covering the thyroid adenoma breakpoint cluster 19q13.4 (TBPC19) [4]. In none of the nuclei of either of the two cases or of the negative control signal patterns corresponding to 19q13.4 rearrangements were detected (Figure 4A,B). As a positive control cytologic smears of adenoma S1016 were used. In this case, FISH revealed 83.5% positive nuclei (one pair of colocalized signals, two separate signals) (Figure 4C). G-banding analysis of this case revealed an apparently balanced translocation t(2;19)(p13;q13.4) (Figure 4D).

## Discussion

Whereas adenomas and goiters belong to highly prevalent lesions of the thyroid not only in iodine-deficient regions malignant thyroid tumors are rare. Both papillary and follicular carcinomas of the thyroid are supposed to originate from the thyroid epithelium. At least for the follicular carcinomas a possible continuum from thyroid adenomas to follicular carcinomas has been proposed [21]. This idea is at least supported by a particular subtype of follicular tumors i.e. those characterized by a PAX8-PPARγ fusion due to a chromosomal rearrangement between chromosomal bands 2q13 and 3p25. As witnessed by the results of several studies, this abnormality can be found in follicular adenomas as well as in carcinomas [22-24]. However, the question arises whether or not other genetic subtypes of thyroid adenomas may predispose to malignant transformation. Recently, we have been able to demonstrate that translocations of chromosomal band 19q13.4, a frequent genetic alteration in thyroid adenomas, activate two miRNA clusters on the long arm of chromosome 19 [5]. Of these, C19MC is the largest human miRNA cluster encoding 59 mature miRNAs [14,25]. The cluster is primate specific and its members are abundantly expressed in embryonic stem cells (ESC) and in the placenta where strong expression is even detectable at term. The cluster is apparently controlled by an adjacent CpG island [6]. Maternal methylation leads to an almost exclusive expression of the paternal allel which, however, also becomes epigenetically silenced in the embryoblast while the “original”, i.e. ESC-like, methylation pattern persists in the placenta. Relatively little is known about the function of that cluster and putative targets of its miRNAs. Although different miRNAs of C19MC have been shown to promote proliferation, invasion and other oncogenic functions [16,26,27], some have also been linked to tumor suppressive properties [28-30]. Overall there are thus conflicting data as to the functions of C19MC miRNAs [for summary see [20]] that so far give no sufficient explanation as to how these miRNAs contribute to the development of thyroid adenomas.

The second cluster affected by the 19q13.4 translocation seen as recurrent clonal chromosomal abnormalities in thyroid adenomas is much smaller. miR-371-3 only encodes five miRNAs and has orthologues in most mammalian species. This cluster is also abundantly expressed in embryonic stem cells and its re-expression in malignant tumors has been associated with invasiveness in a couple of studies [16-19]. These latter findings led us to suggest that activation of this cluster in thyroid adenomas may predispose these lesions to malignant transformation. If this hypothesis holds true one would expect this cluster to be overexpressed in subgroups of thyroid carcinomas from thyroid follicular epithelium. Interestingly, of 114 malignant thyroid tumors no lesion displayed an expression pattern akin to that seen in thyroid adenomas with 19q13.4 translocations, i.e. the simultaneous overexpression of genes of both clusters. In contrast, two tumors were found to overexpress miR-372 and miR-371a-3p but did neither abundantly express miR-520c-3p and miR-519a-3p nor displayed 19q13.4 rearrangements after FISH using appropriate DNA probes.

There is little doubt that chromosomal translocations involving 19q13.4 are causally associated with the development of a subgroup of thyroid adenomas. Though highly recurrent, the mechanism by which they contribute to tumorigenesis is unknown. By these translocations involving varying translocation partners two gene clusters of miRNAs whose members have been associated with malignant growth are reactivated. Nevertheless, from the results of the present study as well as from data obtained on adenomas [5] no evidence exists that in thyroid tumors overexpression of both clusters resulting from 19q13.4 rearrangements characterizes lesions with the capability to invade surrounding tissue, i.e. to undergo malignant transformation. Thus, their function in adenomagenesis still remains to be elucidated.

## Conclusion

Upregulation of the two miRNA clusters C19MC and miR-371-3 does not seem to characterize a major pathway of malignant transformation of the thyroid epithelium.

## Methods

### Tissues

FFPE tumor samples used for the analyses were retrieved from the archive of one of the authors (K. J.).

### RNA isolation

RNA was isolated using the High Pure RNA Paraffin Kit (Roche Applied Science, Mannheim, Germany) as outlined below with modifications for the recovery of small RNAs according to Tang et al. [31].

100 μl tissue lysis buffer, 16 μl 10% SDS and 40 μl proteinase K were added to three 5 μm sections of FFPE tissue and the mixture was incubated for 15 min at 65°C and another 15 min at 80°C. After addition of 325 μl binding buffer the mixture was added to 350 μl absolute ethanol preliminarily put onto a High Pure filter tube. After centrifugation for 20 s at 8,000 × g the flow-through was discarded and the filter tube centrifuged again with the same conditions to dry the filter tube membrane. The membrane was then washed consecutively with 500 μl wash buffer I, 500 μl wash buffer II and 300 μl wash buffer II followed by centrifugation for 20 s at 8,000 × g, respectively, and a terminal centrifugation for 2 min at full speed to remove excess buffer. A mix consisting of 4 μl DNase, 6 μl 10x DNase buffer, and 50 μl aqua bidest was put onto the membrane and incubated for 30 min at 37°C. The DNase treatment was followed by another three wash steps with wash buffer I and II and centrifugations as described above. The RNA was then eluted by putting 50 μl elution buffer onto the membrane, incubating for 1 min and centrifuging for 1 min at 8,000 × g. RNA concentration was measured on a Biophotometer (Eppendorf, Hamburg, Germany).

### Quantitative real-time PCR for detection of miR-372, miR-371a-3p, miR-520c-3p, and miR-519a-3p

Reverse transcription of miR-372, miR-371a-3p, miR-520c-3p, miR-519a-3p, and RNU6B (for normalization) was conducted using the TaqMan MicroRNA Reverse Transcription Kit (Life Technologies Corporation, Carlsbad, USA) according to the manufacturer's instructions.

Real-time PCR was conducted on an ABI 7300 Real-time PCR system using TaqMan MicroRNA Assays (Life Technologies cat. no. 4427975; assay names hsa-miR-371-3p, hsa-miR-372, hsa-miR-520c-3p, hsa-miR-519, RNU6B) and the TaqMan Universal PCR Mastermix (Life Technologies) according to the manufacturer's instructions.

### Fluorescence in situ hybridization

To detect or exclude rearrangements of 19q13.4, cases with elevated expression were further analyzed by FISH using a dual-color, break-apart probe of the thyroid breakpoint cluster 19q13.4 (TBPC19) (PanPath, Budel, Netherlands) on FFPE tissue sections as described previously [32]. For pretreatment of 4 μm tissue sections digestion with a pepsin ready-to-use solution (DCS, Hamburg, Germany) was performed at 37°C for 2 × 45 min (case KS 100) and 1 × 30 min (case KS 13), respectively.

Images were captured with a high performance CCD-camera (Visitron Systems, Puchheim, Germany) and edited with FISH View (Applied Spectral Imaging, Migdal HaEmek, Israel). 200 non-overlapping nuclei from at least four different areas of the tumor were scored. As a control 100 non-overlapping nuclei from non-neoplastic thyroid tissue outside the tumor area of case KS 100 were scored. Two fusion signals indicate a non-rearranged thyroid breakpoint cluster 19q13.4, whereas one fusion signal along with a single green and a single red signal indicate a rearrangement of the thyroid breakpoint cluster 19q13.4. As a positive control, FISH was performed on cytological smears of S1016 as described previously [33].

## Abbreviations

ATC: Anaplastic thyroid carcinoma; C19MC: Chromosome 19 microRNA cluster; ESC: Embryonic stem cells; FFPE: Formalin-fixed, paraffin-embedded; FISH: Fluorescence in situ hybridization; FTC: Follicular thyroid carcinoma; miRNA: microRNA; MTC: Medullary thyroid carcinoma; OTC: Oncocytic thyroid carcinoma; PTC: Papillary thyroid carcinoma; RQ: Relative quantification; TBPC19: Thyroid adenoma breakpoint cluster 19q13.4 dual-color break-apart rearrangement probe.

## Competing interests

No competing interests declared.

## Authors' contributions

VR and JB designed the study and analyzed the data. IF and JB drafted the manuscript and analyzed the data. JWD and IF carried out the expression analyses. JWD analyzed the data and helped to draft the manuscript. ND carried out the fluorescence in situ hybridization. BR did the cytogenetic analyses. KJ and DK provided the samples and carried out the histological examinations. All authors read and approved the final manuscript.
